# Purification and Characterization of Double-Stranded Nucleic Acid-Dependent ATPase Activities of Tagged Dicer-Related Helicase 1 and its Short Isoform in *Caenorhabditis elegans*

**DOI:** 10.3390/genes11070734

**Published:** 2020-07-01

**Authors:** Taishi Kobayashi, Takuro Murakami, Yuu Hirose, Toshihiko Eki

**Affiliations:** Molecular Genetics Laboratory, Department of Applied Chemistry and Life Science, Toyohashi University of Technology, Toyohashi, Aichi 441-8580, Japan; kobatai0122@gmail.com (T.K.); murakami.takuro.eo@tut.jp (T.M.); hirose@chem.tut.ac.jp (Y.H.)

**Keywords:** antiviral RNA interference, *Caenorhabditis elegans*, Dicer-related helicases, isoforms, nucleic acid-dependent ATPase

## Abstract

The Dicer-related helicases (DRHs) are members of a helicase subfamily, and mammalian DRHs such as retinoic acid-inducible gene-I (RIG-I), are involved in antiviral immunity. *Caenorhabditis elegans* DRH-1 and DRH-3 play crucial roles in antiviral function and chromosome segregation, respectively. Although intrinsic double-stranded RNA-dependent ATP-hydrolyzing activity has been observed in the recombinant DRH-3 protein prepared from *Escherichia coli*, there are no reports of biochemical studies of the nematode RIG-I homolog DRH-1. In this study, the secondary structure prediction by JPred4 revealed that DRH-1 and DRH-3 had distinct N-terminal regions and that a 200-amino acid N-terminal region of DRH-1 could form a structure very rich in α-helices. We investigated expressions and purifications of a codon-optimized DRH-1 with four different N-terminal tags, identifying poly-histidine (His)-small ubiquitin-like modifier (SUMO) as a suitable tag for DRH-1 preparation. Full-length (isoform a) and a N-terminal truncated (isoform b) of DRH-1 were purified as the His-SUMO-tagged fusion proteins. Finally, the nucleic acid-dependent ATPase activities were investigated for the two His-SUMO-tagged DRH-1 isoforms and His-tagged DRH-3. The tagged DRH-3 exhibited dsRNA-dependent ATPase activity. However, detectable dsRNA dependency of ATPase activities was not found in either isoform of tagged DRH-1 and a tag-free DRH-1 (isoform a) treated with SUMO protease. These observations suggest that DRH-1 and its short isoform have no or poor nucleic acid-dependent ATPase activity, unlike DRH-3 and mammalian DRHs.

## 1. Introduction

Extensive studies of mammalian natural immunity have revealed the important roles of retinoic acid inducible gene-I (RIG-I), melanoma differentiation-associated gene 5 (MDA5), and laboratory of genetics and physiology 2 (LGP2) in triggering an antiviral response associated with the induction of interferon and other cytokines [1,2]. These proteins, also known as RIG-I-like receptors (RLRs), belong to the Dicer-related helicases (DRHs) subfamily of the helicase superfamily [3,4]. Both RIG-I and MDA5 sense the presence of viral nucleic acids and transduce the viral infection signals downstream in the antiviral response pathways, and it has been suggested that LGP2 may regulate RLR signaling. These mammalian DRHs share the central helicase domain and the C-terminal RIG-I domain for nucleic acid binding. Purified DRHs exhibit ATP-hydrolyzing abilities in vitro that are required by or stimulated by ligand RNAs [5,6,7]. Two mammalian DRHs other than LGP2 have two N-terminal caspase recruitment domains (CARDs), which function as an effector domain for RLR signal transduction [3]. 

Unlike the cytokine-based mammalian immune systems, the antiviral immunity in plants and invertebrates relies on RNA interference (RNAi)-based antiviral systems. Besides the pseudogene *drh-2*, two genes (*drh-1* and *drh-3*) encoding DRHs have already been identified in the *Caenorhabditis elegans* genome. Over a decade ago, our research group comprehensively identified the helicase-like genes in *C. elegans* and investigated the effects of X-ray irradiation on nematodes, where the helicase gene function was abolished by systematic feeding RNAi [8]. *D2005.5*, encoding a novel RNA helicase-like protein, was successfully identified and was later formally named *drh-3*. Nakamura et al. demonstrated that the gene product DRH-3 is a unique DRH in nematodes that is essential for of germ cell production and chromosome segregation, unlike other DRHs [9]. On the other hand, several genetic studies have shown that DRH-1 is a RIG-I homolog that plays a role in antiviral immunity in nematodes [10,11,12,13,14,15]. Two DRHs were co-purified with the nematode Dicer (DCR-1) in a previous proteomic study [16], and it was clarified that these two DRHs act in distinct RNAi pathways. It is currently thought that DRH-1 and DRH-3 function in the biosynthesis of primary small interfering RNA (siRNA) from viral dsRNA and in the amplification of secondary siRNA in nematode RNAi pathways, respectively [3]. These two functionally divergent *drh* genes are well conserved in nematodes [17], indicating their physiological importance. It is of particular interest that DRH-3 is a unique DRH that is absolutely essential for nematode reproduction, unlike other DRHs that act in antiviral responses. Therefore, it is crucial to clarify the biochemical differences between these two nematode DRHs to understand the evolution of the functional diversification of DRHs.

Regarding the biochemical studies of nematode DRHs, Matranga and Pyle prepared DRH-3 proteins using a bacterial protein expression system and reported an intrinsic double-stranded (ds) RNA-dependent ATPase activity and RNA binding ability [18]. Additionally, biochemical properties, such as the dsRNA-dependent ATPase activity of mammal RLRs, have been extensively characterized to elucidate the viral dsRNA-triggered molecular functions [1,2]. However, the successful purification or biochemical characterization of the nematode RIG-I homolog DRH-1 has not been reported. 

In this study, we used bioinformatic prediction of the secondary structures of DRH-1 and DRH-3 to elucidate a unique structure rich in α-helices in the N-terminal region of DRH-1. Next, we investigated the tags suitable for the expression and affinity purification of a codon-optimized DRH-1 in *Escherichia coli*, leading to the successful purification of poly-histidine (His)-small ubiquitin-like modifier (SUMO)-tagged DRH-1 and its short isoform b. Finally, we compared the ds nucleic acid-dependent ATPase activities of the purified DRH-1 proteins with those of His-tagged DRH-3.

## 2. Materials and Methods 

### 2.1. Materials

We purchased 30-nucleotide (nt) RNAs and DNAs that were synthesized by Hokkaido Systems Science (Sapporo, Japan). Other synthetic DNAs were obtained from Fasmac (Atsugi, Japan) and Integrated DNA Technologies (Coralville, IA, USA). The codon-optimized *drh-1* gene fragments were synthesized by Life Technologies (Carlsbad, CA, USA). Plasmid vectors, Gateway LR clonase II, and SUMO protease were obtained from Invitrogen (Thermo Fisher Scientific, Waltham, MA, USA). *Dpn* I, KOD FX Neo DNA polymerase, and the KOD-Plus-Mutagenesis kit were from Toyobo (Osaka, Japan). The NucleoSpin Gel and polymerase chain reaction (PCR) Clean-up kits, In-Fusion cloning kits, and NucleoSpin Plasmid EasyPure kits were from Takara Bio (Kyoto, Japan). Peroxidase-conjugated monoclonal antibodies (anti-His (9C11) and anti-glutathione S-transferase (GST)) were from Wako Pure Chemical (Osaka, Japan), and peroxidase-conjugated goat anti-rabbit immunoglobulin G and anti-Smt3 antibody (ab14405) were from Zymed Laboratory (San Francisco, CA, USA) and Abcam (Cambridge, UK), respectively. Reagents for western blotting and Coomassie brilliant blue (CBB) staining were purchased from ATTO (Tokyo, Japan); yeast extracts and triptone from Nacalai Tesque (Kyoto, Japan); ethylenediamine tetraacetic acid (EDTA)-free protease inhibitor cocktail tablets from Roche (Indianapolis, IN, USA); and poly(dA), poly(dT), poly(A), and poly(U) from Pharmacia (Uppsala, Sweden). Molecular weight marker proteins and DNAs were obtained from Bio-Rad (Hercules, CA, USA), Wako Pure Chemicals, Thermo Fisher Scientific, and Nippon Gene (Tokyo, Japan). Other reagents were of biochemical grade and were obtained from Sigma-Aldrich (St. Louis, MO, USA), Wako Pure Chemicals, and Nacalai Tesque. Polyvinylidene fluoride (PVDF) membranes, Bradford dye reagent, acrylamide gel solution, and Mini Macro-Prep High Q columns were obtained from Bio-Rad. Strep-Tactin Superflow agarose, Strep-Tactin Elution Buffer, and Amicon Ultra-4 centrifugal filer units came from Merck Millipore (Burlington, MA, USA), and anti-FLAG M2 affinity resin and FLAG elution buffer were purchased from Sigma. The His Trap HP columns and GSTrap HP columns were obtained from GE Healthcare (Chicago, IL, USA).

### 2.2. Plasmid Construction 

We prepared recombinant plasmids for the bacterial expression of four different forms of N-terminal tagged DRH-1. A pET-SUMO/*drh-1*op plasmid was constructed for expressing the codon-optimized His-SUMO-tagged DRH-1 in *E. coli*. Briefly, the codon-optimized *drh-1*op DNA was amplified using KOD FX Neo DNA polymerase with the pENTR/*drh-1*op plasmid DNA as a template and a pair of polymerase chain reaction (PCR) primers (Table 1). Then, the PCR product was fractionated using 1% agarose gel electrophoresis and purified with a NucleoSpin Gel and PCR Clean-up kit. The vector DNA was amplified from a Champion™ pET SUMO vector using inverse PCR with the indicated primer set (Table 1), followed by overnight digestion with *Dpn* I and purification, as already described. The *drh-1*op DNA was cloned into the vector using an In-Fusion cloning kit to obtain the pET-SUMO/*drh-1*op plasmid. 

The pET-Strep-SUMO/*drh-1*op and pET-FLAG-SUMO/*drh-1*op plasmids were constructed for the expression of Strep-SUMO- and FLAG-SUMO-tagged DRH-1, respectively. A His-tag sequence in the pET-SUMO/*drh-1*op plasmid was replaced by a Strep-tag or FLAG-tag sequence using inverse PCR, followed by self-ligation using the indicated primer sets (Table 1) and a KOD-Plus-Mutagenesis kit. The pDEST15/*drh-1*op was constructed from a Gateway LR reaction using a pDEST15 vector and a pENTR/*drh-1*op plasmid for the expression of GST-tagged DRH-1. 

A pET-SUMO/*drh-1*op isoform b plasmid was constructed to express a His-SUMO-tagged short isoform of DRH-1 *in E. coli* using inverse PCR with a primer set (Table 1) and pET-SUMO/*drh-1*op DNA as a template, followed by self-ligation using a KOD-Plus-Mutagenesis kit. All constructed plasmids were transformed to *E. coli* DH5α cells, and the resultant transformants were cultured in Luria–Bertani (LB) medium with appropriate antibiotics (20 μg/mL kanamycin for pET-SUMO plasmids and 100 μg/mL ampicillin for pDEST15 plasmids) and purified using a NucleoSpin Plasmid EasyPure kit. The nucleotide sequences of the constructed plasmids were determined using custom dideoxy DNA sequencing (Macrogen Japan Corp., Kyoto, Japan) to confirm the sequence correctness.

### 2.3. Preparation of the Tagged Proteins

The constructed protein expression plasmids were transformed into *E. coli* C41(DE3) cells [19]. The resulting transformants were precultured in 10 mL of LB medium containing the appropriate antibiotics at 37 °C with shaking overnight. Then, the cells were inoculated and cultured in a 2 L flask containing 1 L of LB medium with antibiotics at 37 °C with horizontal rotation (100 rpm) in an incubator (RS-20R; Sanki-Seiki, Osaka, Japan) until optical density values of 0.4–0.8 were attained at 600 nm. Protein synthesis was induced in the *E. coli* cells in the presence of 1 mM isopropyl β-D-1-thiogalactopyranoside (IPTG) by further culture overnight at 15 °C with shaking. The subsequent treatments and purifications were performed at 0–4 °C. First, the cells were collected by centrifugation and resuspended in 50 mL of ice-cold buffer A (25 mM HEPES-NaOH, pH 7.5; 100 mM NaCl; 0.01% [v/v] Nonidet P-40 [NP-40]; and 1 mM dithiothreitol [DTT]) supplemented with EDTA-free protease inhibitor cocktail. Then, the cell extracts were prepared by passing through a French press (5620-S; Ohtake-Seisakusho, Tokyo, Japan) three times under a pressure of 1500 kg/cm^2^. Finally, the cell extracts were centrifuged at 40,000 rpm for 40 min in a 70Ti rotor (Beckman Coulter, Indianapolis, IN, USA). The resultant supernatant was successively subjected to affinity column chromatography corresponding to each N-terminal tag as described below.

For purification of His-SUMO-tagged proteins, the resultant supernatant (50 mL) was loaded at a flow rate of 2 mL/min into a His Trap HP column (1-mL bed) equilibrated with buffer A in an ÄKTA start system (GE Healthcare). The column was washed with 20 mL of buffer A, and then the bound proteins were eluted with a 50-mL linear gradient of 0–0.4 M imidazole and fractionated at 1.5 mL per tube. Purification of GST-tagged DRH-1 was also performed in an ÄKTA start apparatus equipped with a GSTrap HP column (5-mL bed) equilibrated with buffer A. The supernatant was loaded into the column at a flow rate of 5 mL/min. After the column was washed with 100 mL of buffer A, the bound proteins were eluted with a 50 mL linear gradient of 0.0–15 mM glutathione using buffer A and GST elution buffer (50 mM HEPES-NaOH, pH 8.0; 100 mM NaCl; 15 mM glutathione; and 1 mM DTT). Purification of the His-tagged DRH-3 protein was performed as already described, except for the preparation of the cell extracts. The cells containing His-tagged DRH-3 were suspended in 50 mL buffer A with protease inhibitors and disrupted by seven repeated treatments using a BioNeb Cell Disruption System (105A BN3025; Glas-Col, Terre Haute, IN, USA) under N_2_ gas at a pressure of 0.9 MPa. 

The supernatant (50 mL) containing Strep-SUMO-tagged DRH-1 was mixed in a rotatory mixer (20 rpm) with 2 mL of Strep-Tactin Superflow agarose at 4 °C for 1 h. The resin was packed in a column (1 mL bed, Bio-Rad) and washed with 10 mL of Strep wash buffer (100 mM Tris-HCl, pH 8.0; 150 mM NaCl; 10 mM EDTA; and 1 mM DTT). The proteins were eluted with 7 mL of StrepTactin elution buffer and fractionated at 1 mL per tube. The purification of FLAG-SUMO-tagged DRH-1 was performed according to the instructions provided. Briefly, 50 mL of the supernatant was loaded into an anti-FLAG M2 affinity column (1 mL bed) and equilibrated with tris-buffered saline (TBS) with 1 mM DTT at a flow rate of 2 mL/min. The proteins were eluted with 7 mL of FLAG elution buffer after washing the column with 20 mL of TBS plus 1 mM DTT. The eluted fractions (1 mL per tube) were quickly mixed with 25 μL of 1 M Tris-HCl (pH 8.0) for neutralization. 

The His-SUMO-tagged proteins (DRH-1 isoforms a and b) were further purified using anion-exchange column chromatography in preparation for use in ATPase assays. Eluted fractions containing tagged proteins were collected and dialyzed twice against 1 L of dialysis buffer A (25 mM HEPES-NaOH, pH 7.5; 50 mM NaCl; 0.5 mM EDTA; 0.01% [v/v] NP-40; 1 mM DTT; and 10% [v/v] glycerol) at 4 °C for 2 h, and this was repeated once overnight. The dialyzed fraction was loaded into a Bio-Rad Mini Macro-Prep High Q column (5 mL bed) equilibrated with buffer B (25 mM HEPES-NaOH, pH 7.5; 0.01% NP-40; and 1 mM DTT) containing 50 mM NaCl in a BioLogic LP system (Bio-Rad). After washing the column with 20 mL of 50 mM NaCl-containing buffer B, the proteins were eluted with a 50 mL linear gradient of 50–450 mM NaCl and collected in fraction of 1.5 mL. The fractions containing the eluted proteins were collected and dialyzed against 1 L of dialysis buffer B (25 mM HEPES-NaOH, pH 7.5; 100 mM NaCl; 0.5 mM EDTA; 0.01% [v/v] NP-40; 1 mM DTT; and 20% [v/v] glycerol), as already described, and they were further concentrated using centrifugation with an Amicon Ultra-4 ultrafiltration unit. All fractions and aliquots of purified proteins were stored at −80 °C until use.

### 2.4. Protein Analysis

Protein concentrations were determined using the Bradford assay (Bio-Rad), with bovine serum albumin as a standard. Aliquots (4.5 μL) of the proteins in the fractions and purified proteins were subjected to 10% sodium dodecyl sulfate-polyacrylamide gel electrophoresis (SDS-PAGE) in a slab-gel electrophoresis apparatus (ATTO). Two protein gels (8 × 9 cm) were prepared: One gel was subjected to protein visualization by CBB staining in EzStain Aqua solution (ATTO), and the other gel was used for western blotting. The CBB-stained gel image was acquired using a scanner (GT-X800; EPSON, Suwa, Japan). The tagged proteins in the gels were electroblotted to a PVDF membrane using a PoweredBlot Ace blotting apparatus (ATTO) and were detected by western blotting with the antibody corresponding to each tag. To detect His- and GST-tagged proteins, the membrane was blocked with EzBlock Chemi (ATTO), incubated overnight at room temperature with 10,000-fold diluted peroxidase-conjugated anti-His or anti-GST monoclonal antibody, and then washed with EzTBS solution (ATTO). The tagged proteins were visualized with the EzWestLumi plus reagent (ATTO). The blocked membrane was incubated overnight with 2000-fold diluted anti-Smt3 antibody to detect the FLAG-SUMO- and Strep-SUMO-tagged proteins. After washing with EzTBS, the membrane was incubated for 2 h with 2,500-fold diluted peroxidase-labeled goat anti-rabbit IgG and washed again with EzTBS. The membrane was treated for the detection of the tagged proteins, as already described. The chemiluminescent membrane images were acquired using an LAS-4000 mini image analyzer (GE Healthcare).

### 2.5. Nucleic Acid-Dependent ATPase Assay

The nucleic acid-dependent ATPase activity of the purified proteins was determined using a QuantiChrom ATPase/GTPase Assay Kit (BioAssay Systems, Hayward, CA, USA), according to the manufacturer’s instructions. This system quantifies the amounts of free phosphates generated from ATP using the ATPase reaction by measuring the absorbance at 620 nm (*A*_620_) of the dark green color of malachite green reagent that forms when phosphates are generated. According to the manufacturer’s instructions, the ATPase reactions (20 μL in total volume) were performed in 2× assay buffer with 1 mM ATP and 500 nM purified proteins in the presence and absence of 400 nM ds nucleic acids at 37 °C for 0, 10, 20, and 30 min in triplicate. Then, 80 μL of the reagent included in the kit was added, the samples were incubated at 25 °C for 30 min, and 50 μL aliquots were transferred to a 384-well Nunc microplate (cat no. 242765, Thermo Fisher Scientific). After centrifugation of the plate at 1000 × *g* for 1 min in an EX-125 centrifuge (Tomy Digital Biology, Tokyo, Japan), the *A*_620_ values were determined using an Infinite M1000 microplate reader (Tecan, Männedorf, Switzerland). The dsRNA and dsDNA were prepared by annealing the synthesized complementary 30 nt RNAs and DNAs (5’-AGUGCAUCUCCUUCCCUCCUUUCCUUCUGG-3’, and 5’-CCAGAAGGAAAGGAGGGAAGGAGAUGCACU-3’ and 5’-AGTGCATCTCCTTCCCTCCTTTCCTTCTGG-3’ and 5’-CCAGAAGGAAAGGAGGGAAGGAGATGCACT-3’), respectively. Poly(A/U) and poly(dT/dA) (average length, 220 nt) were each prepared by annealing single-strand polymers. Complementary nucleic acids were heated in TE buffer at 98 °C for 2 min and then cooled at room temperature for annealing. The amount of phosphates formed in each ATPase reaction was calculated using a calibration curve obtained from the phosphate standard included in the kit. 

To assay tag-free DRH-1, His-SUMO-tagged DRH-1 (isoform a) was treated with or without 2 U SUMO protease in 50 μL of the assay buffer at 4 °C overnight, and then the ATPase activities of the resultant preparations were measured in duplicate, as described above.

### 2.6. Sequence Analysis

The amino acid sequences of DRHs were obtained from the National Center for Biotechnology Information (https://www.ncbi.nlm.nih.gov/). The secondary structures of the 600-amino acid N-terminal sequences of DRH-1 and DRH-3 were predicted using the JPred4 server [20]. Multiple alignments of the amino acid sequences of DRHs in the Supplementary Figures S1 and S2 were performed by ClustalW v2.1 in the Genetyx MAC package v.19 (Genetyx Co., Tokyo).

## 3. Results and Discussion

### 3.1. Domains and Predicted Secondary Structures in C. elegans DRHs

**Figure 1:**
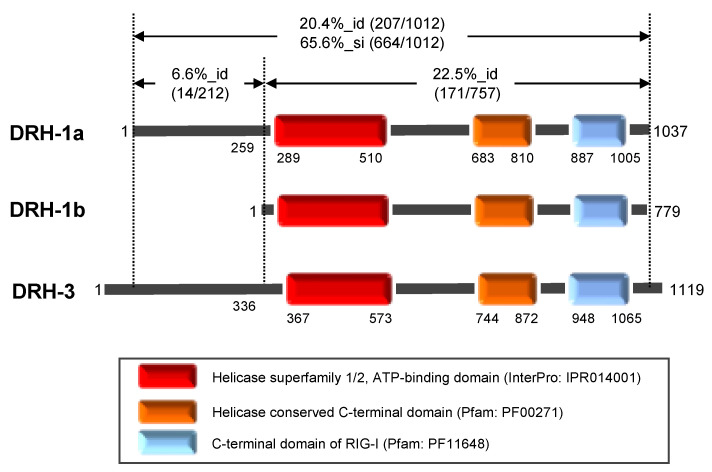
The *Caenorhabditis elegans* Dicer-related helicase (DRH) proteins prepared in this study. The schematic structures of DRH-1 isoforms a and b and of DRH-3 are shown. Numbers indicate the amino acid residue positions. DRH-1 isoform b lacks the N-terminal 258-amino acid region that is present in isoform a. The three domains conserved in DRHs and the positions of their amino acid residues are indicated by the box outlines. The percent identity (%_id) and/or similarity (%_si) in the entire region as well as the N-terminal and the central-to-C-terminal regions between DRH-1a and DRH-3 are indicated as the number of identical or similar residues in the total number of residues in the region.

Two DRHs, namely, DRH-1 and DRH-3, play distinct roles in *C. elegans* [3]. Unlike for the *drh-3* (*D2005.5*), two transcripts are synthesized from *drh-1* (*F15B10.2*). According to the current WormBase (WS276) (https://wormbase.org), the long transcript, F15B10.2a, encodes the DRH-1 isoform a, comprising 1037 amino acid residues (119.2 kDa; NCBI accession number NP_501018), and the short transcript, F15B10.2b, encodes the DRH-1 isoform b, lacking the N-terminal region of isoform a (779 amino acid residues, 89 kDa; NP_001368373), respectively. The short transcript has been confirmed by transcript evidence. The primary structures of DRH-1 and DRH-3 have a 20.4% sequence identity and a 65.6% sequence similarity, and the sequence homology in the N-terminal region (i.e., 6.6% sequence identify) was markedly low compared with that in the remaining region (Figure 1). Both DRH-1 and DRH-3 share conserved domains with other DRHs: the helicase superfamily 1/2 ATP-binding domain (InterPro: IPR014001), the helicase conserved C-terminal domain (Pfam: PF00271), and the C-terminal domain of RIG-I (Pfam: PF11648) at the central and the C-terminal regions (Figure 1). Previous studies of RIG-I demonstrated that these helicase-related domains are involved in ATP hydrolysis, and the C-terminal RIG-I domain binds with dsRNA to trigger a conformational change of RIG-I [2,3,4]. The helicase-like region in *C. elegans* DRH-1 (i.e., amino acid position 259–810) contains motifs I–VI, which are conserved in many helicases and nucleic acid-dependent ATPases and shared with DRH-3, DRH-1 and DRH-3 of other *Caenorhabditis* spp., and human DRHs (Supplementary Figure S1). Matranga and Pyle [18] reported that purified DRH-3 expressed in *E. coli* demonstrated intrinsic dsRNA-dependent ATPase activity, suggesting that DRH-1 has a dsRNA-dependent ATP-hydrolyzing ability.

**Figure 2:**
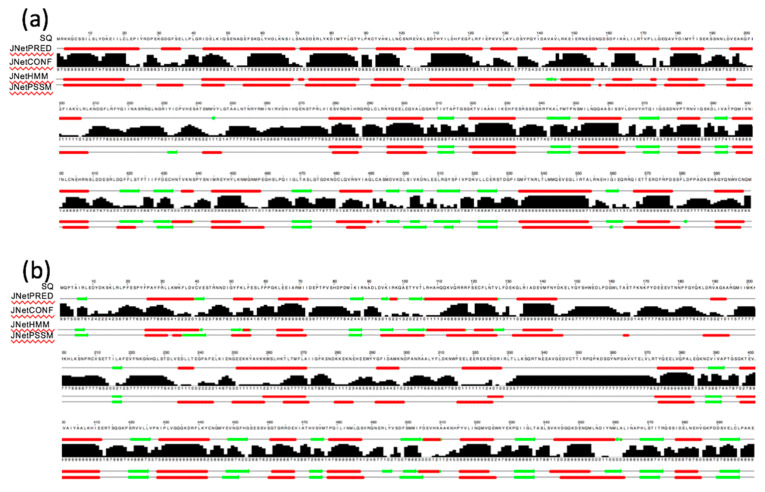
Predicted secondary structures of the 600-amino acid N-terminal sequences of *Caenorhabditis elegans* Dicer-related helicases (DRHs). The 600-amino acid N-terminal sequences of DRH-1 (**a**) and DRH-3 (**b**) were analyzed using the JPred4 server [20] to obtain the predicted secondary structures. SQ: amino acid sequence and the position from the N-terminus. Red tubes and green arrows in the predictions of JNetPRED (the consensus prediction), JNetHMM (hidden Markov model profile-based prediction), and JNetPSSM (position-specific scoring matrix-based prediction) indicate α-helices and ꞵ-sheets, respectively. JNetCONF indicates the confidence estimate for the prediction. High values and histograms shown in black indicate high confidence.

On the other hand, the N-terminal amino acid sequences of nematode DRHs are unique and have no CARDs, unlike RIG-I and MDA5, suggesting that these regions are likely involved in distinct functions of the two nematode DRHs. Additionally, a conserved N-terminal region of DRH-1 is shared in six *Caenorhabditis* species (Supplementary Figure S2). To elucidate the structural features of these regions, the secondary structures were predicted for the 600-amino acid N-terminal regions of DRH-1 and DRH-3 using the JPred4 server [20]. The predicted secondary structures of the two DRHs were distinct. Interestingly, the N-terminal 200 amino acid region of DRH-1 could form a structure that was significantly rich in α-helices (Figure 2a, top line), but this structure was not predicted in the corresponding region of DRH-3 (Figure 2b, top line). Guo et al. demonstrated that the N-terminal domain of DRH-1 (isoform a) is required for suppressing viral propagation in vivo [12], suggesting an important role for this region in antiviral RNAi. Although the molecular functions of the N-terminal regions of DRH-1 (isoform a) and DRH-3 remain unknown, these unique regions may be involved in the diverged cellular functions of the two nematode DRHs via possible protein–protein interactions with other proteins.

### 3.2. Investigation of Tags Suitable for Expression and Purification

**Figure 3:**
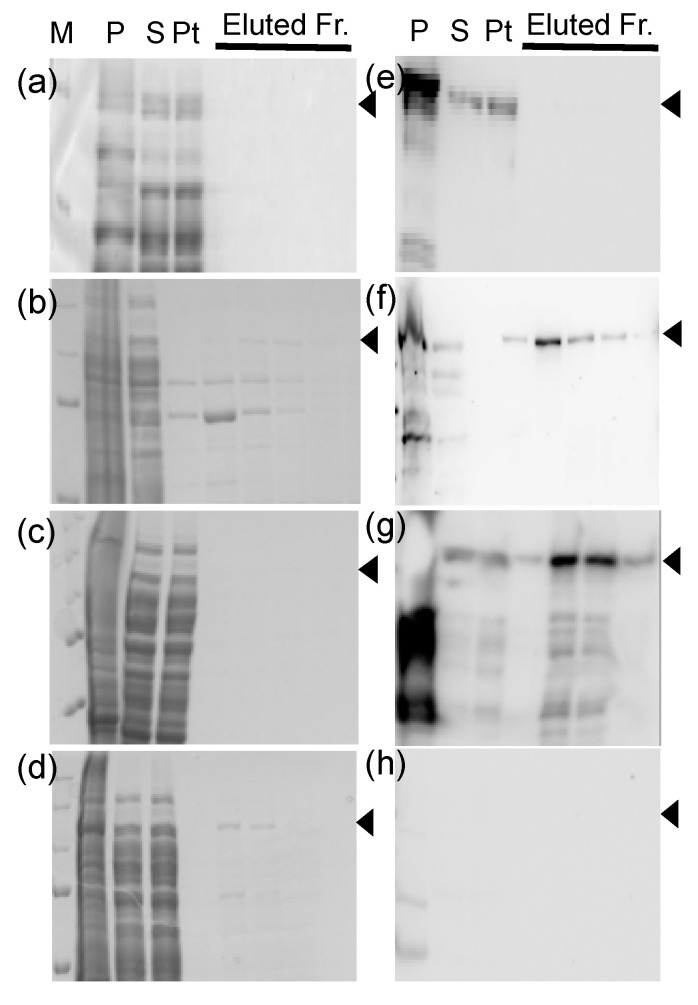
Analyses of four tagged Dicer-related helicase (DRH)-1 proteins purified using affinity-column chromatography. The 4.5 μL aliquots of the stepwise eluted fractions from affinity column chromatography (fractionated from left to right) for the GST- (**a**,**e**), poly-histidine (His)-small ubiquitin-like modifier (SUMO)- (**b**,**f**), Strep-SUMO- (**c**,**g**), and FLAG-SUMO- (**d** and **h**) tagged DRH-1 were subjected to 10% sodium dodecyl sulfate-polyacrylamide gel electrophoresis and visualized using Coomassie brilliant blue stain (**a**–**d**) and western blotting (**e**–**h**). The proteins in an equal volume of aliquots of the pellet suspensions in 10 mL buffer A (P), the supernatants (S) loaded into a column, and the pass-through fractions (Pt) from each purification were also analyzed. Precision Plus Protein Standards (Bio-Rad) were used as molecular weight markers (M). Arrowheads indicate the predicted molecular weights of the tagged proteins: GST-tagged DRH-1, 145.2 kDa; His-SUMO-tagged DRH-1, 131.3 kDa; Strep-SUMO- and FLAG-SUMO-tagged DRH-1, 131.4 kDa.

We first attempted to characterize the biochemical properties of DRH-1 by purifying His-tagged DRH-1 expressed in *E. coli*, as affinity purification had succeeded for an N-terminal His-tagged DRH-3 using the *E. coli* protein expression system, as described in the Materials and Methods section. However, due to the very low levels of protein expression (data not shown), we failed to obtain His-tagged DRH-1. Next, the codons of *C. elegans drh-1* cDNA were optimized for protein expression in *E. coli*. Additionally, four different N-terminal tags were tested (i.e., GST-, FLAG-SUMO-, Strep-SUMO-, and His-SUMO-tags) to identify suitable tags for efficient expression and affinity purification of the codon-optimized DRH-1. The GST and SUMO tags were tested in terms of enhancing the folding and solubility of the expressed proteins, and the FLAG, Strep, and His tags were tested in terms of increasing the purity of affinity-purified proteins. Each tagged DRH-1 underwent affinity purification from the cell extract prepared from a 1-L culture of *E. coli* cells after inducing protein expression. The proteins in the column-eluted fractions, the pellet fractions, the supernatants, and the column pass-through fractions were fractionated using SDS-PAGE and visualized by successive CBB staining and western blots (Figure 3). The GST-tagged DRH-1 was not obtained, because most tagged proteins were precipitated in the pellet (Figure 3a,e). Of the three tagged proteins containing a SUMO tag, the yield of Strep-SUMO-tagged DRH-1 was significantly poorer, even though the expressed proteins were detected by western blotting (Figure 3c,g). The bands of the FLAG-SUMO- and His-SUMO-tagged DRH-1 were detected in the eluted fractions in CBB-stained gels (Figure 3b,d); however, only the latter bands were detected by western blotting (Figure 3f,h). In addition, the FLAG-tagged proteins were eluted from a column using acidic elution buffer (pH 3.5), which could inactivate possible ATPase activity by causing protein denaturation. Thus, we decided to use the His-SUMO-tag for further DRH-1 purification.

### 3.3. Preparations of His-SUMO-Tagged DRH-1 and its Short Isoform

**Figure 4:**
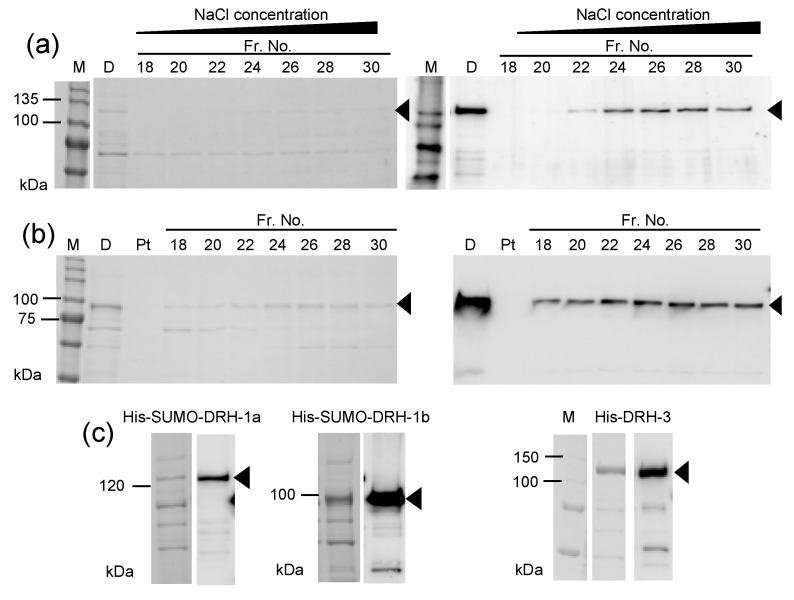
Protein analyses of the poly-histidine (His)-small ubiquitin-like modifier (SUMO)-tagged Dicer-related helicase (DRH)-1 and DRH-1 isoform b eluted from anion-exchange column chromatography. The affinity-purified His-SUMO-tagged DRH-1 (**a**) and DRH-1 isoform b (**b**) were further purified after dialysis using anion-exchange column chromatography. The proteins in 4.5 μL aliquots of the indicated fractions, the dialyzed proteins loaded into the column (D), and the pass-through fraction (Pt) were analyzed using 10% sodium dodecyl sulfate-polyacrylamide gel electrophoresis (SDS-PAGE) followed by Coomassie brilliant blue (CBB) staining (left) and western blotting with peroxidase-labeled anti-His-tag antibody (right). The pooled peak fractions (numbers 24–30) of both His-SUMO-tagged DRH-1 and DRH-1 isoform b were concentrated using ultrafiltration, and then analyzed as already described (CBB staining, left and western blotting, right in panel **c**). The affinity-purified and concentrated His-tagged DRH-3 was also analyzed using 12% SDS-PAGE followed by CBB staining and western blotting in panel **c**. WIDE-VIEW Pre-stained Protein Size Marker III (Wako Pure Chemicals), MagicMark XP Western Protein Standard (Thermo Fisher Scientific), and Precision Plus Protein Standards (Bio-Rad) were used as molecular weight markers (M) for CBB staining and Western blotting in panels (**a**, **b** and **c**), respectively. Arrowheads indicate the predicted molecular weights of the tagged proteins: His-SUMO-tagged DRH-1, 131.3 kDa, His-SUMO-tagged DRH-1 isoform b, 101.1 kDa, and His-tagged DRH-3, 132 kDa.

The *drh-1* gene is known to act in antiviral RNAi in *C. elegans,* and the current WormBase suggests that long and short transcripts were synthesized from the gene *in vivo*. The observation reported by Guo et al. strongly suggested that the N-terminal region of long DRH-1 (isoform a) is necessary for antiviral function [12]. What could the physiological role of the small isoform b, without the N-terminal region of long DRH-1 be? In mammalian DRHs, LGP2 lacks the CARD-containing N-terminal regions of RIG-I and MDA5 and plays a regulatory function in antiviral immunity [7,21,22]. Therefore, isoform b might be involved in nematode antiviral RNAi, similar to LGP2. His-SUMO-tagged DRH-1 isoforms were prepared (Figure 1) to characterize and compare the biochemical properties of the two isoforms. The synthesis of tagged proteins was induced in *E. coli* C41(DE3) cells in the presence of 1 mM IPTG at 15 °C overnight. The supernatants from ultra-centrifuged cell extracts were subjected to nickel affinity chromatography, and the eluted proteins were successively purified using anion-exchange column chromatography. The proteins in the eluted fractions were analyzed using SDS-PAGE followed by staining with CBB and western blotting with anti His-tag antibody (Figure 4). Discrete bands of DRH-1 isoform b (Figure 4b) and faint bands of DRH-1 (Figure 4a) were detected in the CBB-stained gels. The molecular weights of the detected bands were almost in agreement with the values calculated from their amino acid sequences (i.e., 131.3 and 101.1 kDa for His-SUMO-tagged isoforms a and b, respectively). Peak fractions containing the proteins (fractions 24–30) were pooled, dialyzed, and concentrated for subsequent ATPase assay (Figure 4c). The amounts of purified His-SUMO-tagged DRH-1 isoforms a and b were 225 and 800 μg, respectively. Approximately 2.7 mg of the affinity-purified His-tagged DRH-3 was obtained from a comparable culture and used for the assay (Figure 4c). Although it is difficult to directly compare the amounts of purified DRH-1 and DRH-3 because of their different purification steps (i.e., one step for DRH-3 and two steps for DRH-1), the yield of purified DRH-1 was almost 10-fold lower than that of DRH-3. Most of the expressed DRH-1 was solubilized in *E. coli,* such that there were no detectable tagged proteins in the pellet (Figure 4), indicating apparently low levels of DRH-1 synthesis compared with that of DRH-3 in *E. coli*. Additionally, the amount of purified isoform b was approximately 4-fold larger than the amount of DRH-1 isoform a. These data likely suggest that the N-terminal region of DRH-1, with a structure predicted to be rich in α helices, suppresses the synthesis of DRH-1 isoform a in *E. coli*.

### 3.4. Nucleic Acid-Dependent ATPases of the Purified DRH-1 Proteins

**Figure 5:**
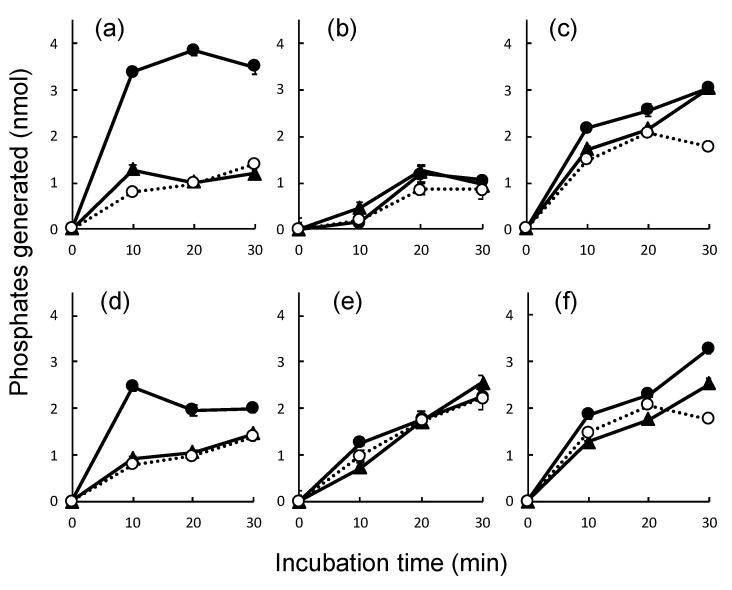
Double-stranded (ds) nucleic acid-dependent ATPase assays of the tagged Dicer-related helicase (DRH)-1, DRH-1 isoform b, and DRH-3. ATPase activities for 500 nM of the poly-histidine (His)-tagged DRH-3 (**a**,**d**), His-small ubiquitin-like modifier (SUMO)-tagged DRH-1 (**b**,**e**), and His-SUMO-tagged DRH-1 isoform b (**c**,**f**) were measured in the absence (open circles on dotted lines) or presence of dsDNA (closed triangles) and dsRNA (closed circles) using a QuantiChrom ATPase/GTPase Assay Kit, as described in the Materials and Methods section. Additions of 400 nM of short 30-nucleotide ds oligonucleotides and long ds polynucleotides (poly(A/U) and poly(dA/dT)) were made in the reactions shown in (**a**–**c)** and (**d**–**f**), respectively. The reactions were performed in triplicate at 37 °C for indicated periods, and the phosphate amounts generated in the reactions were plotted with the standard deviations.

Finally, the nucleic acid-dependent ATPase activities of the prepared proteins were examined via a colorimetric assay using a QuantiChrom ATPase/GTPase Assay Kit. ATPase reactions containing 500 nM of proteins were performed at 37 °C for 0, 10, 20, and 30 min in the presence or absence of 30 nt dsRNA and dsDNA. The resultant phosphates bound to malachite green were measured colorimetrically. The dsRNA-dependent ATPase activity of His-tagged DRH-3 was successfully detected, as reported previously using untagged DRH-3 (Figure 5a). On the other hand, a relatively low level of ATPase activity was detected in the tagged DRH-1 protein, but ds nucleic acid-dependency of ATP hydrolysis was not observed (Figure 5b). The tagged DRH-1 isoform b also exhibited ATP-hydrolyzing activities, but detectable ds nucleic acid dependency was not found (Figure 5c). Another study further tested the effects of long dsRNA and dsDNA in the ATPase reactions with DRH-1 proteins because it is believed that DRH-1 acts in processing virus-derived long dsRNAs to primary siRNAs in antiviral RNAi in *C. elegans* [23]. The dsRNA dependency of ATPase activity of DRH-3 was slightly decreased with long ds ribopolymer poly(A/U) compared with that with short dsRNA (Figure 5d). Although ATPase activity was observed in both DRH-1 isoforms, no significant dsRNA dependency of ATPase activity was observed (Figure 5e,f). Although it is possible that a portion of the ATPase activity observed was derived from bacterial ATPases contaminating the DRH-1 proteins (Figure 5c), significant dsRNA-dependent ATPase activities in addition to those activities were not detected in the DRH-1-containing reactions. Thus, the results indicate that DRH-1 has no or poor dsRNA dependency for the ATPase activity. 

**Figure 6:**
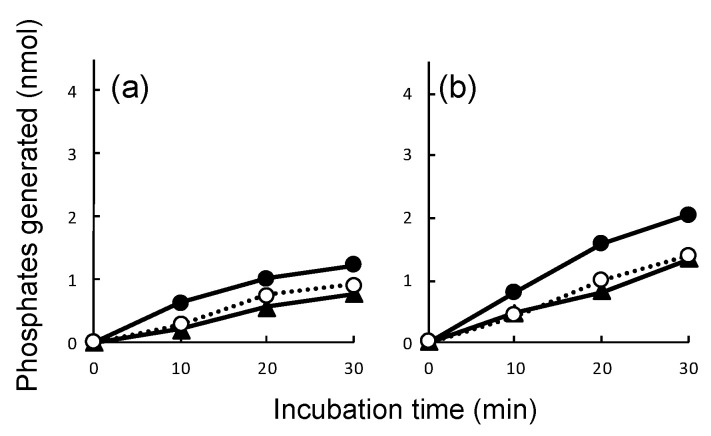
Double-stranded nucleic acid-dependent ATPase assays of Dicer-related helicase (DRH)-1 treated with and without small ubiquitin-like modifier (SUMO) protease. Poly-histidine (His)-SUMO-tagged DRH-1 was digested with and without SUMO protease (**a** and **b**, respectively). The ATPase activity of approximately 500 nM of DRH-1 was measured in the absence (open circles on dotted lines) or presence of dsDNA (closed triangles) and dsRNA (closed circles) using a QuantiChrom ATPase/GTPase Assay Kit, as described in the Materials and Methods section. The reactions were performed in duplicate at 37 °C for the indicated periods, and the phosphate amounts generated in the reactions were plotted.

Several possibilities can be considered to account for the poor dsRNA-dependent ATPase activity of tagged DRH-1. First, the N-terminal His-SUMO tag may sterically inhibit the enzymatic activity. To address the question, we removed the SUMO tag by digesting with or without SUMO protease at 4 °C and assayed their ATPase activities in the presence or absence of dsRNA and dsDNA. Low levels of ATP-hydrolyzing activities with no significant dsRNA dependency were observed in both the tag-free (Figure 6a) and tagged (Figure 6b) states. Thus, possible inhibition of dsRNA-dependent ATPase activity caused by the N-terminal His-SUMO tag can be excluded.

Secondly, the lack of post-translational modifications of the DRH-1 used may affect the enzymatic activity. It is possible that *in vivo* modifications of DRH-1 are required to fully exhibit intrinsic ATPase activity because the DRH-1 used in this study was prepared in *E. coli*. Although this possibility cannot be entirely excluded, it may be unlikely because the DRH-1 paralog DRH-3 expressed in *E. coli* exhibits significant dsRNA-dependent ATPase activity in our study (Figure 5a) and other studies [18]. Another possibility requiring investigation is that DRH-1 may require other accessory proteins for exhibiting significant dsRNA-dependent ATPase activity.

Currently, the reason why long and short isoforms of DRH-1 do not exhibit significant dsRNA-dependency in ATP hydrolysis remains unknown. Alternatively, some DRHs may act in their pathways without dsRNA-dependent ATPase activities. Bamming and Horvath used several helicase motif mutants to show that type I interferon production mediated by full-length MDA5 and RIG-I is independent of helicase domain catalytic activity [24]. Since Tabara et al. reported that DRH-1 was co-purified with other proteins, including DCR-1, and complexed with RDE-4 *in vivo* [23], DRH-1 may act as a platform protein for a functional complex for processing long dsRNA to primary siRNAs in an ATPase-independent manner.

## Figures and Tables

**Table 1:** Polymerase chain reaction primer sets used for plasmid construction. Lower-case letters in the nucleotide sequences correspond to the nucleotide sequences for the tags.

Constructs	Primers	Nucleotide Sequence (5’–3’)
pET-SUMO/*drh-1*op	drh1op-pET-SUMO15-IFF	GAACAGATTGGTGGTATGCGTAAAAAACAGTGTAGCAGC
	drh1op-pET-SUMO15-IFR	TACCTAAGCTTGTCTTTATGCTTCACGAATCAGGTTCAC
	pET-SUMO-5’-invR	ACCACCAATCTGTTCTCTGTGAGCC
	pET-SUMO-3’-invF	AGACAAGCTTAGGTATTTATTCGG
pET-Strep-SUMO/*drh-1*op	pET-SUMO_invR_half Strep tail	ctgcgggtggctccaGCTGCTGCCCATATGTATATC
	pET-SUMO_invF_half Strep tail	gacgatgacaaaCTGGTGCCGCGCGGCAGCGCT
pET-FLAG-SUMO/*drh-1*op	pET-SUMO_invR_half FLAG tail	gacgatgacaaaCTGGTGCCGCGCGGCAGCGCT
	pET-SUMO_invF_half FLAG tail	ttcgaaaaaggcgcgCTGGTGCCGCGCGGCAGCGCT
pET-SUMO/*drh-1*op isoform b	pET-SUMO-5’-invR	ACCACCAATCTGTTCTCTGTGAGCC
	drh1op_259-invF	ATGATTAACATCCGCGTGGATAAC

## Supplementary Materials

The following are available online at https://www.mdpi.com/2073-4425/11/7/734/s1, Figure S1: Multiple alignment of helicase-like domains of nematode and human DRHs. Figure S2: Multiple alignment of DRH-1 sequences of six *Caenorhabditis* species.
