# Supplementary material for: Purification and Characterization of Double-Stranded Nucleic Acid-Dependent ATPase Activities of Tagged Dicer-Related Helicase 1 and its Short Isoform in Caenorhabditis elegans
Source: Genes (Basel). 2020 Jul 1;11(7):734. doi: 10.3390/genes11070734 (PMC7397212; doi:10.3390/genes11070734)
Supplement: Supplementary file 1 [file genes-11-00734-s001.pdf]

Supplementary Materials

Article

Purification and characterization of double-stranded nucleic acid-dependent ATPase activities of tagged Dicer-related helicase 1 and its short isoform in *Caenorhabditis elegans*

Taishi Kobayashi, Takuro Murakami, Yuu Hirose and Toshihiko Eki*

Molecular Genetics Laboratory, Department of Applied Chemistry and Life Science, Toyohashi University of Technology, Toyohashi, Aichi 441-8580, Japan ; kobatai0122@gmail.com (T. Kobayashi); murakami.takuro.eo@tut.jp (T. Murakami); hirose@chem.tut.ac.jp (Y. Hirose); eki@chem.tut.ac.jp (T. Eki)

*Correspondence: eki@chem.tut.ac.jp; Tel.: +81-532-44-6907 (T.E.)

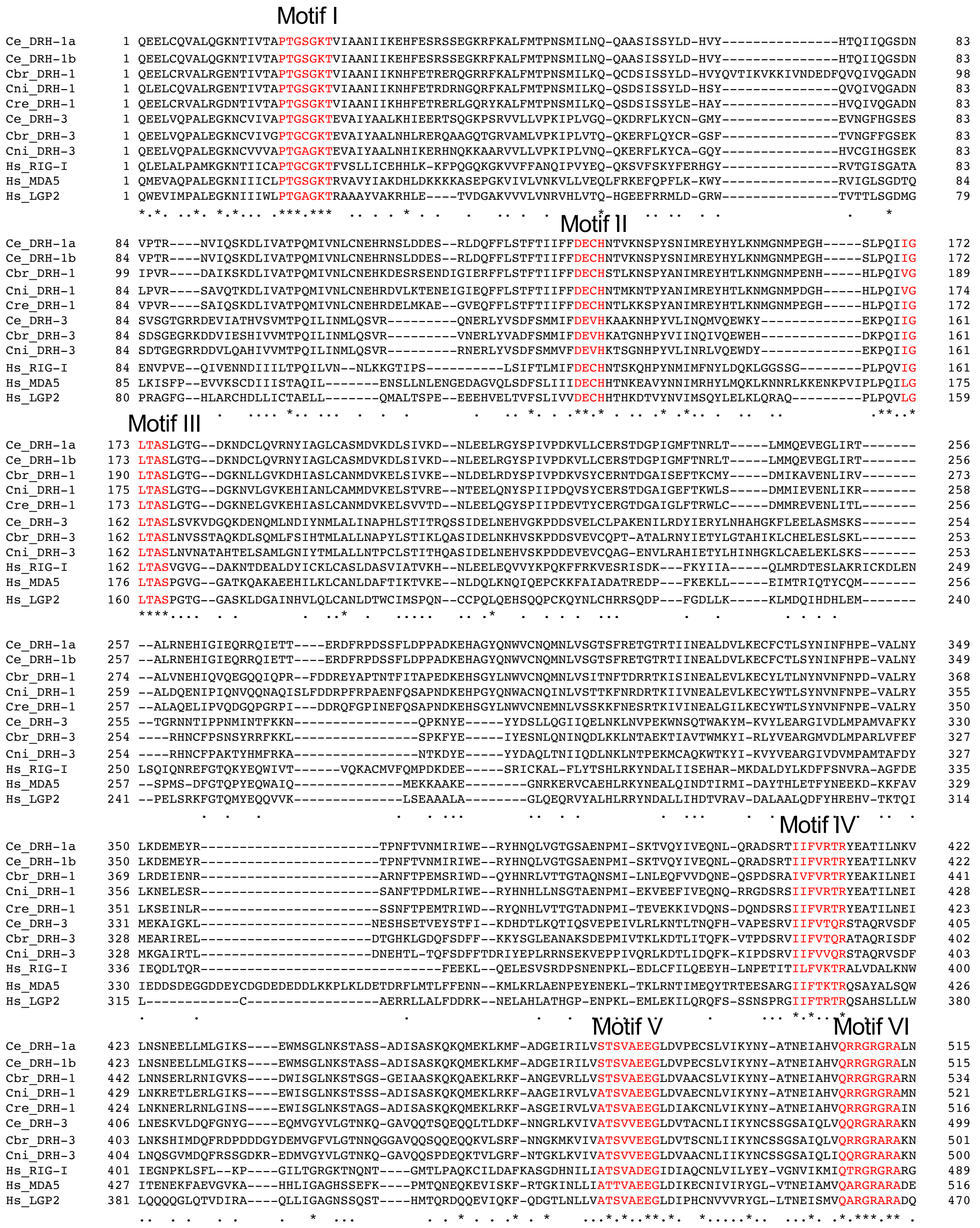

Figure S1. Multiple alignment of helicase-like domains of nematode and human DRHs. The amino acid sequences corresponding to the helicase-conserved motifs I to VI are indicated in red. Identical and similar amino acid residues are shown under the aligned sequences by asterisks and dots, respectively. Abbreviations of *Caenorhabditis* species (the GenBank accession number): Ce, *C. elegans* (Isoform a, NP_501018 and isoform b, NP_001368373); Cbr, *C. brenneri* (EGT53499); Cni, *C. nigoni* (PIC33712); Cre, *C. remanei* (XP_003096885); Hs, *Homo sapiens* (RIG-I, NP055129; MDA5, NP071451.2; LGP2, NP077024).

```
Ce_DRH-1a      1 -MRKKQCSSILSLYDKEIILCLEPIYRDPEKGDGFSELLPLGRIDELKIQSEN---AQEFSKQLYHDLKNSILSNA-DDERLYKDIMTYLQTYLPKCTVH    95
Cbr_DRH-1      1 MVRKKECAALVRLYEREIIRCLEPIFREPDRGEHFEELLTRGRIEELIGESDN---EMDFAKKLFVELTSSPLSLA-DDERLYKDVMAYNHANLRNSAVH    96
Cla_DRH-1      1 MVRKKQCAALVKLYDREITRCLEPIYQEPDKGEYFEELLSLGRIEELIGEFEN---AVDFSKKLFQELSESPLTRDRDEERLYKNVMTYLQACLPGSNVH    97
Cre_DRH-1      1 MVRKKQCAALVKLYGREITRCLEPVYQEPEKGEYFEELLSLGRIEELIGEFEN---AVDFSKKLFQELSESPLTRD-DEERLYKNVMTYLQACLPGSNVH    96
Cbg_DRH-1      1 MVRKKQCAALVKLYDREIIRCLEPIYREPEKGEHFDELLSRGRIEELIGEYED---TTSFSSQLFHELQESPISKA-DDERLYKSVMSFLQANFTDSHVH    96
Cni_DRH-1      1 MVRKKQCAALVKLYDREIIRCLEPIYREPEKGEHFEELLSRGRIEELIGEYEDPPNAKGFSSQLFHELQESPISKA-DDERLYKSVMSFLQANFTDSPVH    99

Ce_DRH-1a     96 KLLNCSNREVKLSDFHYILDHFEGFLRFIEPKVVLAYLDSYP-QYIDAVAVLR--KEIER--NEEDNQDSDFIKKLILRTVPLLGEQAVYDIMYTISEKS   190
Cbr_DRH-1     97 RLMSCSDKSIIRSEFCKILRNLDNFLRFLNPTALLGFLGSYPQHYEDLVRVFNEWRNTE----ENEEDNQENMKKAILKMVPLFGEFAVYDIMYSIHEHT   192
Cla_DRH-1     98 KLLKCSDKTMRRCQFSTILNNLDGFLRYSDPETILRYLDCYP-HYKDVVVALR--REIEQ-NRDDETEDEDFIKKLILRTVPMLGESSAYDVMFSIHENT   193
Cre_DRH-1     97 KLLKCSDRTMRRSQFSTILNNLDGFLRYSDPETILRYLDCYP-HYTDVVIALR--REIEQ-NRNDETEDEDFIKKLILRTVPMLGESSAYDIMFSIHENT   192
Cbg_DRH-1     97 KLLKCSDRTMRMCQYGLILNHLDGFINYMDPNEVLTYLDSYP-QYQDIVRELR--RETERIVPEEQQQNTEFLKKSILRIVPLLGESSAYDVMYAIYDKP   193
Cni_DRH-1    100 KLLKCSDRTMRMCQFGLILNHLDDFINYMDPNAVLTYLDFYP-QYQDIVRELR--RETERMEQEQQQQNTEFLKKSILRIVPLLGESSAFDVMYAIYNNP   196

Ce_DRH-1a    191 SNNLDVEAKQFIAKVLRLKNDGFLRFY------QIINASRRQLNGRIYICPVHESATEMMVYLGTAALNTNRYRMINIRVDNIVQENSTPRLVIESVRQR   284
Cbr_DRH-1    193 TNDNRQEAKSFIEQLLHLKEGEFRRYYGIFHFSDSINADRLQRNGQMYICPIHESATEMLVYIGSPNFNTNRYRMINIRHDNIQPEDSVQRLVIQSVRHR   292
Cla_DRH-1    194 SNNLNEEAKTFIENVLQLKRGGFKAFY------EFISADRRQCNGRIYICPIHESATEMLVRL--PEFNDSRYRMINIRYDNIQAEESVPRLVIESVRHR   285
Cre_DRH-1    193 SNNLNEEVKTFIENVLQLKRGGFKAFY------EFISADRRQCNGRIYICPIHESATEMLVRL--PEFNDSRYRMINIRYDNIQAEESVPRLVIESVRHR   284
Cbg_DRH-1    194 SNNLNEEARNFIEKILRLRPGSFDAFY------REVSDDRRRFNGNIFICPINEIATEMIARI--PEVNRHRYRMINIRYDNIQSEPPVPRLIVESVRNR   285
Cni_DRH-1    197 SNNLNEEARAFIEKILRLKPGSFRSFY------REVSDDRRRFNGNIFICPINEIATEMIARL--PAVNRHRYRMINIRYDNIQSEPPVPRLIVESVRNR   288

Ce_DRH-1a    285 IHRQRQLCLRNYQEELCQVALQGKNTIVTAPTGSGKTVIAANIIKEHFESRSSEGKRFKALFMTPNSMILNQQAASISSYLDHVY---------------   369
Cbr_DRH-1    293 IDQQRQLCLRGYQEELCRVALRGENTIVTAPTGSGKTVIAANIIKNHFETRERQGRRFKALFMTPNSMILKQQCDSISSYLDHVYQVTIKVKKIVNDEDF   392
Cla_DRH-1    286 IHLQRQLCLRGYQEELCRVALRGDNTIVTAPTGSGKTVIAANIIKHHFETRERLGQRYKALFMTPNSMILKQQSDSISSYLEHAY---------------   370
Cre_DRH-1    285 IHLQRQLCLRGYQEELCRVALRGDNTIVTAPTGSGKTVIAANIIKHHFETRERLGQRYKALFMTPNSMILKQQSDSISSYLEHAY---------------   369
Cbg_DRH-1    286 IHLQRQLCLRRYQLELCQVALRGENTIVTAPTGSGKTVIAANIIKNHFETRDRNGQRFKALFMTPNSMILKQQSDSISSYLDHSY---------------   370
Cni_DRH-1    289 LHMQRQLCLRRYQLELCQVALRGENTIVTAPTGSGKTVIAANIIKNHFETRDRNGQRFKALFMTPNSMILKQQSDSISSYLDHSY---------------   373

Ce_DRH-1a    370 HTQIIQGSDNVPTRNVIQSKDLIVATPQMIVNLCNEHRNSLDDES--RLDQFFLSTFTIIFFDECHNTVKNSPYS---------------NIMREYHYLK   452
Cbr_DRH-1    393 QVQIVQGADNIPVRDAIKSKDLIVATPQMIVNLCNEHKDESRSENDIGIERFFLSTFTIIFFDECHSTLKNSPYA---------------NIMREYHTLK   477
Cla_DRH-1    371 HVQIVQGADNVPVRSAIQSKDLIVATPQMIVNLCNEHRDELMKAE--GVEQFFLSTFTIIFFDECHNTLKKSPYA---------------NIMREYHTLK   453
Cre_DRH-1    370 HVQIVQGADNVPVRSAIQSKDLIVATPQMIVNLCNEHRDELMKAE--GVEQFFLSTFTIIFFDECHNTLKKSPYA---------------NIMREYHTLK   452
Cbg_DRH-1    371 QVQIVQGADNLPVRNAVQTKDLIVATPQMIVNLCNEHRDVLKTENEIGIEQFFLSTFTIIFFDECHNTMKNTPYASECFPTIPVIMINFSDIMREYHTLK   470
Cni_DRH-1    374 QVQIVQGADNLPVRSAVQTKDLIVATPQMIVNLCNEHRDVLKTENEIGIEQFFLSTFTIIFFDECHNTMKNTPYA---------------NIMREYHTLK   458

Ce_DRH-1a    453 NMGNMPEGHSLPQIIGLTASLGTGDKNDCLQVRNYIAGLCASMDVKDLSIVKDNLEELRGYSPIVPDKVLLCERSTDGPIGMFTNRLTLMMQEVEGLIRT   552
Cbr_DRH-1    478 NMGNMPENHHLPQIVGLTASLGTGDGKNLLGVKDHIASLCANMDVKELSIVKENLDELRDYSPIVPDKVSYCERNTDGAISEFTKCMYDMIKAVENLIRV   577
Cla_DRH-1    454 NMGNMPEGHHLPQIIGLTASLGTGDGKNELGVKEHIASLCANMDVKELSVVTDNLEELQGYSPIIPDEVIYCERGTDGAIGLFTRWLCDMMREVENLITL   553
Cre_DRH-1    453 NMGNMPEGHHLPQIIGLTASLGTGDGKNELGVKEHIASLCANMDVKELSVVTDNLEELQGYSPIIPDEVTYCERGTDGAIGLFTRWLCDMMREVENLITL   552
Cbg_DRH-1    471 NMGNMPDGHHLPQIVGLTASLGTGDGKNVLGVKEHIANLCAMMDVKELSTVRENTEELQNYSPIIPDR--------------------EILNRNDEFL--   548
Cni_DRH-1    459 NMGNMPDGHHLPQIVGLTASLGTGDGKNVLGVKEHIANLCAMMDVKELSTVRENTEELQNYSPIIPDQVSYCERSTDGAIGEFTKWLSDMMIEVENLIKR   558

Ce_DRH-1a    553 ALRNEHIGIEQRRQIETT----ERDFRPDSSFLDPPADKEHAGYQNWVCNQMNLVSGTSFRETGTRTIINEALDVLKECFCTLSYNINFHPEVALNYLKD   648
Cbr_DRH-1    578 ALVNEHIQVQEGQQIQPR--FDDREYAPTNTFITAPEDKEHSGYLNWVCNQMNLVSITNFTDRRTKISINEALEVLKECYLTLNYNVNFNPDVALRYLRD   675
Cla_DRH-1    554 ALAQEHIPVQDGQPGRPI---DDRQFGPINEFQSAPNDKEHSGYLNWVCNEMNLVSSKKFNESRTKIVINEALGILKECYWTLSYNVNFNPEVALRYLRS   650
Cre_DRH-1    553 ALAQELIPVQDGQPGRPI---DDRQFGPINEFQSAPNDKEHSGYLNWVCNEMNLVSSKKFNESRTKIVINEALGILKECYWTLSYNVNFNPEVALRYLKS   649
Cbg_DRH-1    549 SFYEENIPIQNVQQNVQNSLFDDRPYRPAENFQSAPNDKEHPGYLNWACNQMNLVCTAKFNRDGTKIIINEALEVLKECYWTLSYNVNFNPEVALRYLKS   648
Cni_DRH-1    559 ALDQENIPIQNVQQNAQISLFDDRPFRPAENFQSAPNDKEHPGYQNWACNQINLVSTTKFNRDRTKIIVNEALEVLKECYWTLSYNVNFNPEVALRYLKN   658

Ce_DRH-1a    649 EMEYRTPNFTVNMIRIWER---------------YHNQLVGTGSAENPMISKTVQYIVEQNLQRADSRTIIFVRTRYEATILNKVLNSNEELLMLGIKSE   733
Cbr_DRH-1    676 EIENRARNFTPEMSRIWDQ---------------YHNRLVTTGTAQNSMILNLEQFVVDQNEQSPDSRAIVFVRTRYEAKILNEILNNSERLRNIGVKSD   760
Cla_DRH-1    651 EINLRSSNFTPEMARIWDR---------------YQNHLVTTGTADNPMITEVEKKIVDQNSDQNDSRSIIFVRTRYEATILNEILNKNERLSNLGINSE   735
Cre_DRH-1    650 EINLRSSNFTPEMTRIWDR---------------YQNHLVTTGTADNPMITEVEKKIVDQNSDQNDSRSIIFVRTRYEATILNEILNKNERLRNLGINSE   734
Cbg_DRH-1    649 ELESRSANFTHHMSRIWESKFQFLLLLLCIEISGYHNHLLNSGTAENPMIEKVEQFIVDQNEQRGDSRSIIFVRTRYEATILNEILNKRETLERLGIKSE   748
Cni_DRH-1    659 ELESRSANFTPDMLRIWER---------------YHNHLLNSGTAENPMIEKVEEFIVEQNQRRGDSRSIIFVRTRYEATILNEILNKRETLERLGIKSE   743

Ce_DRH-1a    734 WMSGLNKSTASSADISASKQKQMEKLKMFADGEIRILVSTSVAEEGLDVPECSLVIKYNYATNEIAHVQRRGRGRALNSECVLITNSIALRDQESNNRDK   833
Cbr_DRH-1    761 WISGLNKSTSGSGEIAASKQKQAEKLRKFANGEVRLLVSTSVAEEGLDVAACSLVIKYNYATNEIAHVQRRGRGRARNSTCVLITNSVALRDQESSNRDK   860
Cla_DRH-1    736 WISGLNKSTAASADISASKQKQMEKLRKFASGEIRVLVATSVAEEGLDIAKCNLVIKYNYATNEIAHVQRRGRGRAINSKCILITNSIPLRDQEGANRDK   835
Cre_DRH-1    735 WISGLNKSTAGSADISASKQKQMEKLRKFASGEIRVLVATSVAEEGLDIAKCNLVIKYNYATNEIAHVQRRGRGRAINSKCILITNSIPLRDQEGANRDK   834
Cbg_DRH-1    749 WISGLNKSTSSSADISASKQKQMEKLRKFATGEIRVLVATSVAEEGLDVAECNLVIKYNYATNEIAHVQRRGRGRAMNSTCVLVTNSIPLRDQEGANRDK   848
Cni_DRH-1    744 WISGLNKSTSSSADISASKQKQMEKLRKFAAGEIRVLVATSVAEEGLDVAECNLVIKYNYATNEIAHVQRRGRGRAMNSTCVLVTNSIPLRDQEGANRDK   843

Ce_DRH-1a    834 ESLMSETISLIQNSPAEFRKCVDEESNKIWPRILREDTDKAQKIEEQINRNIVYKIICKKCEAILCTSKDIRSRNTQYLVCDPGFWSLVRKTRLTDEQQA   933
Cbr_DRH-1    861 ENMMNQTLLAIQSNPIAFKDTVAVEIGKIWNRIVREDTERAQHVAEQVNQNILYRIVCKKCEVFLCTNKDVRSRGTQYLVCRPAFWSLVRKIKFSPEE--   958
Cla_DRH-1    836 ENMMNRALLKIQSNPFAFRDAVIAEASNIWNRILREDAENAQRIAEQISQNVTYQILCKKCEVLLCTNWDIRARNTQYLVCRPEFWSLVRKVELSPAE--   933
Cre_DRH-1    835 ENMMNKALLKIQSNPFAFREAVTAEASNIWNRILREDAERAQRIADQISQNVTYHILCKKCEVFLCTNWDIRARNTQYLVCRPEFWSLVRKVELSPAD--   932
Cbg_DRH-1    849 ENMMNQALLKITTNPGAFRDAVMAEIGNIWNRIQREDTERARQIAEQISRNVTYRIVCKKCDVFLCTNSDIRARNTQYLVCRPEFWTLVQKIKLTPGEIA   948
Cni_DRH-1    844 ENMMNQALLKITTNPGAFRDAVMAEIGNIWNRIQREDTERAQQIAEQISRNVTYRIVCKKCDVFLCTNSDIRARHTQYLVCRPEFWTLVKKVELKPEEIA   943

Ce_DRH-1a    934 LIKYNATGS----INCRRENCGLKLGQLIEVNTVDLPCLSALSIVL-----------LVE-GTDKRIIVKKWKN-------ILDKYFTPTEIRQLDVQTM  1010
Cbr_DRH-1    959 VDRSHSTGRLYFQILCRGANCGATLGRLLDINSVELPCLGAEGMFFELIRLYYHVEYYSYRHHQRWNRGKKSNQEMENGM-ILHKFFTPLPIRQLDIQTM  1057
Cla_DRH-1    934 ADRCHSTGK----VKCLGRNCGAILGRLIDINSSELPCLAAEAIVL-----------VDQRNTGQMIPIKKWKQ-------ILSKYFTPVDLRQLDIQKM  1011
Cre_DRH-1    933 ADRCHSTGK----VKCLGRNCGAIIGRLIDMNSSELPCLAAEAIVL-----------VDQRNTSKMITIKKWKQ-------ILSKYFTPVDLRQLDIQRM  1010
Cbg_DRH-1    949 TNKFHSTGM----IKCLGTNCGAILGRLIDVTNTELPCLSAEAIVL-----------INE-HEDKRIQAKKWKKVFSNSETILSDHFTPVEIRQLDIQKM  1032
Cni_DRH-1    944 RNKFHSTGM----IKCLGTNCGAILGRMIDVTNTELPSLTAEAIVL-----------INE-NEDKRIQVKKWKK-------ILSDHFTPVDIRQLDIQKM  1020

Ce_DRH-1a   1011 RDADQARTPMVFEHHANGEVVNLIREA                                                                          1037
Cbr_DRH-1   1058 RDASQNRAPINFEVHSNGVIQNINRDA                                                                          1084
Cla_DRH-1   1012 RDAIQARASLNFEFHRNGIIENINREI                                                                          1038
Cre_DRH-1   1011 RDAIHARASLNFEFHRNGIIENINREI                                                                          1037
Cbg_DRH-1   1033 RDANQTRTPLNFELNLNGILQNIIREA                                                                          1059
Cni_DRH-1   1021 RDANQTRTTVNFELNFNGSLQNIIREI                                                                          1047
```

Figure S2. Multiple alignment of DRH-1 sequences of six *Caenorhabditis* species. The accession numbers of DRH-1 sequences are NP_501018 (*C. elegans,* Ce), EGT53499 (*C. brenneri*, Cbr), OZG19204 (*C. latens*, Cla), XP_003096885 (*C. remanei*, Cre), CAP25885 (*C. briggsae*, Cbg), and PIC33712 (*C. nigoni*, Cni), respectively. Identical residues are shown by red boxes.
